# Exploring staff perceptions of the utility of clinician connections when working with emotionally dysregulated clients

**DOI:** 10.1186/s40479-019-0109-0

**Published:** 2019-07-26

**Authors:** Lucy Burke, Mary Kells, Daniel Flynn, Mary Joyce

**Affiliations:** 1 Cork Mental Health Services, Health Service Executive, Psychology Department, Inniscarrig House, Western Road, Cork, Ireland; 2 Cork Mental Health Services, Health Service Executive, Block 2, St Finbarr’s Hospital, Cork, Ireland; 3 National Suicide Research Foundation, Western Gateway Building, University College Cork, Cork, Ireland

**Keywords:** Family connections, Clinician connections, Borderline personality disorder, Dialectical behaviour therapy

## Abstract

**Background:**

Borderline personality disorder (BPD) is considered to be a challenging condition for clinicians to treat. Clinicians routinely working with individuals who experience severe emotional dysregulation often do not receive appropriate training and support to work with this client group. This article describes an intervention, Clinician Connections (CC), which was developed to support practitioners who work with individuals with BPD. CC aims to increase practitioner’s knowledge of BPD, develop a skillset to work with emotionally dysregulated individuals and enhance practitioner’s self-efficacy with regard to working effectively with this client group. The aim of this study is to investigate the perceived utility and acceptability of CC, and identify areas for further development of the intervention.

**Method:**

A seven-hour CC workshop was provided to Emergency Department and community mental health clinicians. Three focus groups were completed following completion of the intervention with 13 clinicians (12 female; 1 male) and were audio recorded. The study utilised a thematic analysis framework.

**Results:**

Six master themes emerged from the focus group data which included 10 subordinate themes. The master themes identified were: the need for training; a new understanding; validation; barriers to applying new skills; overcoming barriers to skill application; and future direction: practical application of skills. Participants reflected on how their new understanding of transactions and their own experiences affects their practice. They also noted improved client interactions and client relationships resulting from the use of validation. While there was an increase in participants’ self-efficacy in working with individuals with BPD, a need for further skills and practice was also highlighted.

**Conclusion:**

The evidence presented here suggests that CC is both beneficial and feasible. Qualitative feedback suggests there is a need for further support in the strengthening and generalisation of skills. Suggestions were made by practitioners regarding potential improvements to the delivery of the workshop. Future research could evaluate the changes made to CC and focus on a quantitative approach to quantify the impact of CC.

## Introduction

Borderline personality disorder (BPD) is a mental health disorder which typically features patterns of cognitive, emotional and behavioural dysregulation [1]. Behaviours which manifest as an attempt to manage emotional dysregulation can often result in crisis presentations to emergency departments (ED) [2]. Such behaviours may include episodes of impulsivity and suicidal behaviour which can be difficult for mental health practitioners to manage [2]. These behaviours can also present challenges which may impact on effective engagement with community mental health teams (CMHTs) [3]. Practitioners working in ED, acute units and on CMHTs are often not trained in a model of psychotherapy to treat BPD [4, 5] and as such, do not necessarily have the training to support patients with severe BPD [3].

BPD is considered to be challenging for clinicians to treat [6] and health staff preconceptions and negative bias about individuals with BPD may further exacerbate difficulties in providing effective treatment. Previous research has suggested that mental health practitioners may hold negative attitudes towards people who have a diagnosis of BPD and who engage in self-harm [7]. Factors which have been found to influence attitudes include service setting, practitioners’ level of experience and the absence of specific training to enhance an understanding of BPD [8]. It has also been reported that mental health practitioners may view suicide and self-harm behaviours as manipulative or attention seeking [9]. In addition, ED staff have reported difficulty in maintaining empathy for individuals with BPD as a result of frequent ED attendance following episodes of self-harm [8]. This can result in BPD becoming a stigmatised disorder [10] and may result in suicide risk being minimised in a population who are already at risk. [11].

As well as the outlined challenges in providing effective treatment to individuals with BPD, these perceived difficulties can also have a negative effect on clinicians’ personal wellbeing. There is evidence to suggest that clinicians who work closely with individuals with BPD often become mentally exhausted, experience depersonalisation and decreased empathy, and question their competency with regard to their ability to work with this patient group [12]. A study which explored stress amongst mental health service providers showed that the three most extreme stressors for practitioners were patients presenting with anger, threats of suicide and suicide attempts [13]. All three are common features of BPD. More specifically, a study which explored stress and burnout in clinicians found that practitioners working with BPD find the experience very stressful [14]. However, it was noted that dialectical behaviour therapy (DBT), while demanding as an intervention to train in, reduced clinician stress when working therapeutically with clients. Clinicians reported feeling supported by the teamwork and supervision components of DBT [14]. In line with such findings, mental health practitioners have also reported that skills training workshops and regular in-house training would be of benefit to support them in working with individuals with BPD [15].

### Structuring the environment

One of the functions of DBT as a treatment for BPD involves structuring the environment; this refers to both the treatment itself and non-treatment environments of patients [16]. Non-treatment environments may refer to situations involving family members or mental health practitioners. There appears to be numerous similarities in the challenges faced by both family members and practitioners caring for or working with individuals with BPD. For example, Hoffman et al. [17] identified that individuals who have a family member with BPD experience feeling overwhelmed and traumatised by behaviours associated with BPD. In addition, there is a high risk of emotional burnout and incidences of highly stressful and chaotic interactions for family members. Although the authors are not aware of any specific interventions which support practitioners working with individuals with BPD, interventions have been developed to support family members. One such example is Family Connections.

### Family connections

Family Connections (FC) is a multi-family, manualised skills training programme offered to families with a member with BPD [18]. It is based on DBT [19] and the stress, coping and adaption model of Lazarus and Folkman [20], which focuses on the strengths, resources, and adaptive abilities of the person. FC was developed with the aims of providing up-to-date psychoeducation about BPD and family functioning, teaching DBT skills, and providing peer support for families [17]. A number of studies conducted on FC have produced promising findings. Hoffman et al. [17] reported significant decreases in levels of burden and grief, and a significant increase in mastery following completion of FC. In a later replication study, they reported similar findings with the addition of a significant decrease in levels of depression reported by participants [21]. Similarly, a reduction in burden and improved relations with the individual have been reported in other studies [22]. Most recently, a study by Flynn et al. found that FC resulted in significant improvements regarding the sense of burden and grief that is experienced by family members when compared to an optimised treatment as usual group [23].

### Interventions for clinicians

As FC has been shown to be effective in ameliorating some of the psychological problems incurred by family members (e.g. burden, grief or depression), it is reasonable to assume that the principles, concepts and skills from FC could be applied to the broader support system and the community of clinicians working with severely emotionally dysregulated patients. With this in mind, the established FC programme for family members was tailored to make it more appropriate for delivery to practitioners working in the ED, acute units and on CMHTS who routinely encounter individuals experiencing severe emotional dysregulation. This adapted programme for practitioners is referred to as Clinician Connections (CC) hereafter. CC aims to increase practitioner’s knowledge of BPD and the five levels of dysregulation which can be experienced by individuals with BPD (emotional, behavioural, interpersonal, self and cognitive dysregulation) [19]. The programme aims to enable mental health practitioners to develop a skillset for working effectively with dysregulated clients and through reflective practice and peer support, decrease their stress levels. Finally, CC aims to enhance practitioner’s self-efficacy with regard to working effectively with individuals with emotional dysregulation.

As no intervention has previously been developed to support health practitioners in non-therapy roles to work with individuals with BPD, we wished to investigate if CC would be of benefit to staff working in the ED, acute units and on CMHTs. The aim of this study is therefore to investigate the perceived utility and acceptability of CC, and explore areas for further development of the intervention for clinicians routinely engaging with individuals experiencing severe emotional dysregulation.

## Method

### Study setting and design

This study was conducted in a public mental health setting in the Republic of Ireland. This study utilised a qualitative research design which employed focus groups to explore staff perceptions of the intervention.

### Intervention

Clinician Connections (CC) is a pilot programme which has been developed and adapted from Family Connections [18]. CC is a 7 hr workshop delivered over 2 days, aimed at mental health practitioners who routinely work with individuals who experience severe emotional dysregulation. The workshop was run over two 3.5 hour sessions, one month apart, to accommodate practitioners’ schedules. There is an initial focus on providing up-to-date psychoeducation about BPD, the biosocial theory and the transactional model [19]. The initial focus included a brief orientation to the evidence base of treatments for BPD; however, CC is derived from DBT and FC which is underpinned by the stress, coping and adaption model of Lazarus and Folkman.

**Fig. 1:**
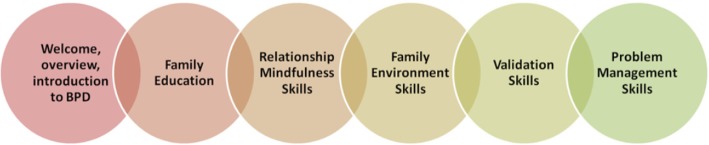
Overview and sequential presentation of the six modules delivered in Family Connections

**Fig. 2:**
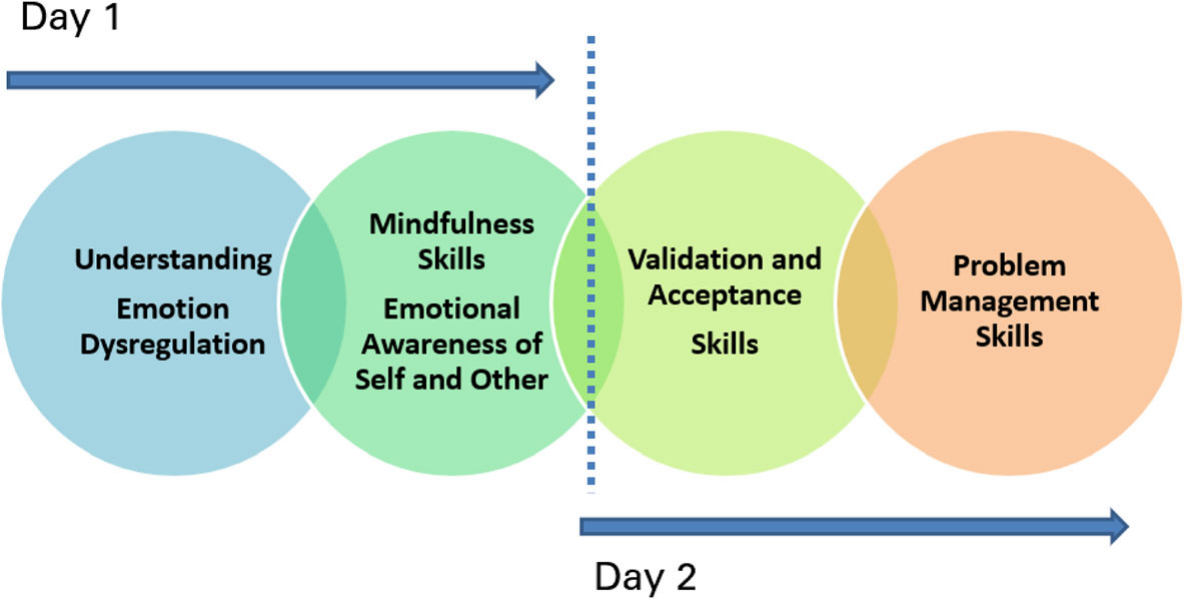
Overview of module content delivered in Clinician Connections by day

Family Connections is comprised of six modules: Introduction to BPD; Family Education; Relationship Mindfulness Skills; Family Environment Skills; Validation Skills; and Problem Management Skills (see Fig. 1). These modules are delivered over 12 two-hour sessions. While parallel versions of each of these modules would arguably have had utility for clinicians, it was not feasible to provide an intervention of this duration in this service. All practitioners who were invited to take part in this programme were qualified mental health professionals employed by the Health Service Executive (HSE). In view of this, a baseline level of knowledge was assumed. Components of FC modules 1 and 2 (Introduction to BPD and Family Education) were combined to form CC module 1 (Understanding Emotional Dysregulation). FC modules 3, 5 and 6 (Relationship Mindfulness, Validation Skills and Problem Management Skills) were retained (see Fig. 2). FC module 4 (Family Environment Skills) was not included. All modules were adapted so that the focus was on the treatment system rather than on the family system. Modules were also condensed. The clinicians delivering the programme are FC leaders and trainers and made content based decisions based on clinical experience.

### Participants

Participants for CC were recruited via an email sent to the relevant services managers who were asked to circulate it to relevant mental health practitioners. A total of 26 practitioners attended CC. All practitioners who attended the CC programme were invited to participate in the research study. Recruitment of participants took place at the end of the second workshop where information about the research study was provided to CC attendees. Individuals who were interested in research participation were invited to sign up to one of three pre-scheduled focus groups. The focus groups were scheduled one month after the second workshop. Of the 26 participants, 13 (12 female; 1 male) partook in the study. Participants were clinicians who worked in the emergency department, and community mental health team workers (medical including non consultant hospital doctors and nurses, and health and social care professionals including occupational therapists and social workers). DBT had been available in the service where the research was conducted for approximately seven years prior to this study.

### Procedure

The protocol of the present study was approved by a statutorily approved ethics committee which operates as part of the national HSE. Focus groups were conducted by the same researcher (one of the authors: LB) to ensure consistency across groups. To aid the validity of the study, the researcher did not attend the CC workshops. Participant information leaflets and consent forms were distributed to participants prior to the start of each focus group. Participants were asked to read the information leaflet and sign the consent form if they wished to proceed with participation in the research study. Each focus group lasted between 35 and 45 minutes. A focus group schedule was utilised and focused on the following: the features of the workshop considered most/least helpful; the need for continuing professional development; whether the skills/information gleaned informed changes in practice; the barriers to skill implementation; further training needs; and practical aspects of the workshop such as duration and location. Each focus group was recorded using a digital audio recorder.

### Analysis

One of the authors (LB) used a thematic analysis framework, as outlined by Braun and Clarke [24], to analyse the data transcribed from the focus groups. Thematic analysis aims to identify and analyse patterns or themes within and across data sets. Braun and Clarke’s six stage approach was applied. See Table 1 below.

**Table 1:** Braun and Clarke’s six stages of thematic analysis

Stages	Action
Familiarisation	Audio files were listened to, transcribed, read and re-read by the researcher
Coding the data	A systematic, line-by-line approach was employed, and codes were assigned to interesting concepts within the data. Illustrative quotes were extracted
Themes	The codes were grouped based on commonalities and subsequent superordinate and subordinate themes were developed
Review of themes	Themes were checked with illustrative quotes and a thematic map was produced
Defining the themes	Themes were succinctly defined, using the language of the participants where possible, to capture the core meaning of the theme
The report	Themes were analysed and reported within the context of service evaluation and provision

## Results

**Fig. 3:**
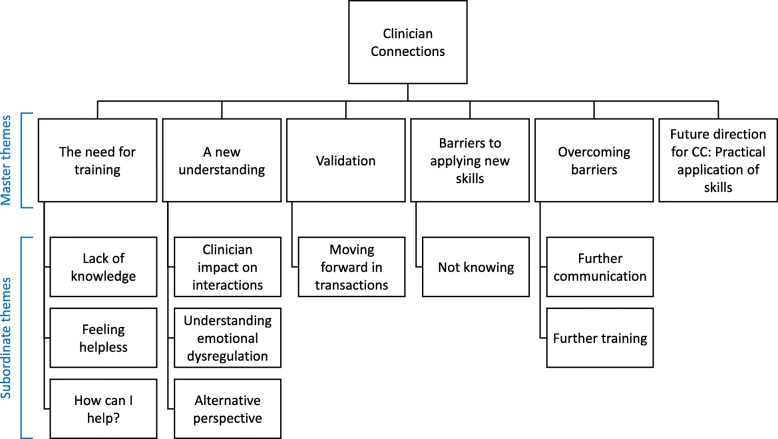
Thematic map outlining six dominant and ten subordinate themes

Six dominant themes and ten subordinate themes were identified in the data (see Fig. 3). These themes are considered to represent the participant’s experience of the CC workshop.

### The need for training

While reflecting on their experiences, there was complete agreement among the participants that there was a need for the CC workshops. Participants identified several areas of need and three subordinate themes were developed which elaborate this further.

#### Feeling helpless

Participants were asked to reflect on the need for continuing professional development (CPD). They expressed experiencing feelings of “*helplessness*” and “*incompetence*” during interactions with clients presenting to the ED or CMHTs with severe emotional dysregulation.

P2: “*The sense of helplessness, getting swallowed up and becoming almost dysregulated yourself.”*

There was a sense of agreement in the groups that as a practitioner, it can be difficult to stay calm and to regulate one’s own emotions when confronted with someone in crisis. This feeling is exacerbated when practitioners feel they don’t have the skills required to support the person presenting in crisis. Participants expressed concerns of *“not knowing what to say”* (P3) and wondering *“what do I do?”* (P5).

#### Lack of knowledge

The theme of having limited knowledge about the DBT programmes offered in the service was apparent across all three focus groups. Participants reported knowing that the DBT programme existed but having little specific awareness of the skills taught in the programme or levels of support offered. Participants stated that they frequently inquire if their clients are using the skills learned on the DBT programmes but noted that they feel they are “*bluffing*” during the interaction:

P8*: “when you tell people to use their DBT skills you are just shooting in the dark.”*

P13: *“asking…but not having a clue what they are”.*

#### How can I help?

Participants wondered how they as practitioners can help clients presenting with emotional dysregulation. All participants expressed the desire to further their skillset to support clients in distress:

P2: *“what can I do right now to help this person?”*

P11: *“I’d love to be able to learn this to help people when we do meet them”.*

### A new understanding

Participants appeared to develop a new understanding of emotional dysregulation and reflected on the fact that the training changed their perception of the person with whom they were interacting. The majority of participants also noted how their developing awareness is changing their practice. Several aspects of the training contributed to this and are further illustrated in the following subordinate themes.

#### Understanding emotional dysregulation

Participants in the three focus groups reflected on the benefits of the psychoeducation component of CC. This component aimed to create a better understanding of the mechanisms involved in emotional dysregulation. Some participants described having previously felt frustrated during challenging interactions with clients presenting to the ED or CMHT. This frustration appeared to occur as a result of not knowing why the client was expressing intense emotions and extreme behaviours. Since attending CC, the practitioners reported developing a greater awareness for the individual and their situation:

P6: *“There’s patterns from their childhood… people can’t help being that way”.*

P3: *“there’s something deeper going on”.*

Participants developed the ability to make sense of the client’s current presentation by considering the underlying mechanisms of how emotional dysregulation develops, and the client’s past history. This in turn impacted upon how practitioners felt about their client. Participants noted that this new understanding of emotional dysregulation and its implications on interactions has had a positive impact on their practice:

P6: *“It takes away the impatience and the lack of empathy you can sometimes have”.*

Participants noted an increase in empathy and a resulting reduction in their previous experience of frustration towards some clients with whom they worked.

#### Clinician impact on interactions

The CC workshop facilitated discussions regarding mindfulness and self-awareness. The clinician’s role in interactions was subsequently discussed across the three focus groups. The majority of participants reflected on their new learning regarding the necessity of being aware of their internal states and how their experience of the client or their own emotions can affect the interaction. Two participants outlined this further:

P4: *“Understanding what is happening to me as well, you tend to blame them but your own facial expressions can make it worse.”*

P8*: “Being mindful of where you are on a given day, that has huge implications for how the interactions can go”.*

In addition to this, several participants expressed a sense of relief when they were reminded during the workshop that they are “*human*” and thus fallible:

P11*: “Learning that you are a human being yourself…it’s good to be mindful of yourself and know that you have a threshold.”*

#### Alternative perspective

CC aimed to foster a deeper understanding of the processes involved in severe emotional dysregulation. After attending CC, practitioners reflected that it changed the way they perceived their clients and difficult interactions:

P9: *“Helped me see a different way of looking at an interaction with my client.”*

P10: *“People aren’t just purposively trying to be difficult.”*

Participants recognised that while it sometimes appeared that the client was behaving in ways which practitioners found challenging, it wasn’t always intentional or directed personally towards the practitioner. A consistent observation across the focus groups was that having an alternative understanding reduced practitioner’s own anxiety about working with the population:

P1: *“There’s less dread meeting the person because you are approaching it differently.”*

### Validation

Validation skills were referred to by all 13 participants as being the most useful component of CC and the most influential in terms of changing their practice:

P12: *“Validation skills encouraged me to stick with it and try harder and not give up with the person.”*

The majority of participants explained previous difficulties they experienced when empathising with and validating people engaged in harmful or destructive behaviours. Following the intervention, they could validate the person’s emotion and experience, without condoning the behaviours:

P7: *“Validation stuck in my mind a lot, how to validate some part of what the person is experiencing even if you don’t agree with a behaviour.”*

Others noted that it changed the way in which they approached their sessions. They previously adhered to agendas and their session goals. Following CC, there is an awareness of letting the person feel heard. This included being flexible with the session: balancing the validation of the person’s emotional experience with change-based strategies.

P5: *“It changes that expectation of getting through the agenda but the most important thing is she is heard.”*

It was acknowledged that this positively affected interpersonal interactions and the therapeutic relationship for both parties.

#### Moving forward in transactions

The subordinate theme of moving forward in transactions emerged from the groups’ discussion of using validation skills. Several participants had been using validation in the month prior to the focus groups. Practitioner’s reflected on how validating the person’s emotional experience helped them progress both their relationship and their planned intervention. They noted that clients who are dysregulated frequently ruminate on a difficult interaction or event and this can be a barrier to therapeutic progress. They noted how validation can be used to overcome this:

P9: *“They can see that you are trying to acknowledge it and then can move forward rather than getting stuck on it”.*

P3: *“It allowed us to move past it a little bit, once they feel ok they know where I’m coming from. Acknowledge and validate it you can move forward rather than just getting stuck in a rut with it.”*

### Barriers to applying new skills

Each focus group was asked to highlight barriers they face in using the skills learned at CC. While lack of confidence was cited as a reason, this appeared to originate from a lack of knowledge regarding the core skills taught in DBT.

#### Not knowing

The majority of participants expressed the desire to learn more specific DBT skills. While their new knowledge has been beneficial, gaps still remain. Several participants stated similar concerns about “*putting a foot in it*” (P4) or “*making things worse.*” (P5). One individual expressed her fear about contradicting the DBT programme and this appeared to be shared by other participants:

P2: “*Not knowing what do to is the main barrier.”*

P8*: “I would be afraid for saying something opposite to what they are learning [on the DBT programme]”.*

Practitioner’s agreed that knowing more about the DBT skills plus DBT structure could alleviate these fears.

### Overcoming barriers to skill application

Practitioners reflected on possible solutions to the barriers discussed and highlighted the need for further training and increased communication.

#### Further training

Practitioners across the three focus groups agreed that further training and practice could help increase their confidence in utilising skills and improve their knowledge base.

P10: *“Having the information can overcome barriers”.*

P6: “*I would like more practical skills maybe roleplays on how to use the skills on a practical level.”*

Knowing the specific skills and how to practice them with an individual experiencing emotional dysregulation was highlighted as a training need. The importance of further training was recognised in terms of having an effect on both practitioner and client well-being:

P7: *“It takes a certain amount of patience and there’s a high level of burn out towards some clients but the training and the discussion part of the training helped with that”.*

#### Further communication

The need for increased communication among peers and across teams was discussed in terms of overcoming barriers. In general, the practitioners found the peer support element of CC reassuring and normalising. One group generated the idea of forming peer support groups at work:

P4: **“***Peer support stuff was reassuring…that others experience these difficulties too.”*

P13: *“Forming support groups among staff to practice the skills.”*

When reflecting on their concerns about “*doing the wrong thing*” (P7) or giving conflicting advice to the DBT programme, several practitioners suggested the need for more communication regarding an individual’s progress in the DBT programme and information about their care plan:

P3: *“More communication on the team about what works for the person”.*

P6: “*To be better linked…who is their telephone contact for phone coaching – whether they are attending the group”.*

The DBT programme offers a number of supports to clients attending the group (e.g. phone coaching outside working hours). Practitioners agreed that knowing more about this process and others like it would be beneficial in terms of providing improved care.

### Future direction for CC: practical application of skills

The predominate theme towards the end of each focus group was the need for further training in the practical skills of DBT. Many participants echoed the desire to obtain tangible skills that can be applied in the moment when clients are in extreme distress:

P13: *“I would like to know more about the practical applications of the skills.”*

P8: *“I’d love to be able to learn this to help people when we do meet them.”*

This learning included the development of a knowledge base of the various concepts used within DBT (e.g. chain analysis) to support clients using their skills:

P12: *“I just know the words but I don’t know what it is, we should probably know how to do it to support them…how to go through it with them.”*

## Discussion

### Findings and recommendations

The aim of this study was to explore practitioner’s perceptions of the utility of CC. CC aimed to provide education and skills to mental health practitioners to work effectively with emotional dysregulation and improve their self-efficacy when working with BPD populations. Thematic analysis identified six themes: the need for further training; a new understanding; validation; barriers to applying new skills; overcoming barriers to skill application; and future direction: practical application of skills. These themes highlight the need for practitioner training to support them in their work with individuals who engage in high risk behaviours. These themes will be discussed in view of potential implications for service provision.

Participants identified the need for ongoing training, reflecting on their experiences of feeling helpless when working with a client with severe emotional dysregulation. One of the primary objectives of CC was to increase practitioner’s knowledge of BPD presentation and to support them in developing skills to work more effectively with the person, whilst being mindful of their own wellbeing. Emerging themes suggest that practitioners perceived they had an increased understanding of BPD and of the transactional model, and how it affects their practice. This qualitative finding appears consistent with existing quantitative findings regarding FC. For example, FC research has noted that information and understanding of BPD has reduced the sense of burden and grief experienced by family members [21] and has improved relations with the individual [22].

Central to building positive relationships within family systems are concepts of reciprocity, mindfulness of self and other and the capacity to validate the other person to help soothe and decrease emotional dysregulation [21]. According to participants in this study, applying this understanding to the wider support network involving mental health practitioners has also resulted in improved interpersonal interactions. Practitioners noted improved relationships with clients since applying validation skills and further noted that it has changed the way in which they approach, prepare and plan for engagement with clients who frequently present with emotional dysregulation. This promising finding warrants more robust quantitative exploration as CC develops. This suggestion of more open engagement by practitioners with their clients mirrors findings in the FC literature which highlighted improved relationships between carers and their relative with BPD [22].

Two further goals of CC were to enhance practitioners’ levels of self-efficacy in managing their own potential to experience emotional dysregulation and to decrease their levels of stress in working with this population. Initial themes indicated that practitioners felt more confident with the skills acquired in CC but described a need to acquire further skills and practice. FC research highlights how participants increase mastery over time [17]. However, direct comparisons between CC and FC are necessarily limited by a number of factors. It is noteworthy that this initial version of CC involved approximately seven contact hours, which is significantly less than the typical contact hours in FC of approximately 24 hours. Similarly, CC was offered over two sessions while FC is offered over 12. Both the contact hours and number of sessions for CC were informed primarily by practical, service constraints.

Given that CC is a brief intervention with practitioners in the form of two workshops one month apart, it is reasonable that practitioners would not yet have fully consolidated and refined their skills. Research suggests that stress and burnout is a significant risk factor for employees and, in particular, mental health practitioners working with clients with severe emotional dysregulation. Work related stress has been shown to have harmful effects on people’s physical and mental health [25]. Employers such as the Health Service Executive in Ireland are becoming increasingly aware of the need to support practitioners in managing work related stress [25]. Service managers need to give due consideration to investment in their practitioner's continuing professional development and allowing time for further peer training sessions or peer reflective practice sessions. It is hoped that this in turn would facilitate practitioners in becoming more confident in skills application for the benefit of themselves and those utilising services.

The dearth of knowledge of the DBT programme structure and the application of DBT skills was highlighted as the main barrier to supporting clients presenting with emotional dysregulation. Basic introductory workshops in DBT and communication across all services were suggested as potential solutions to this. Perhaps in the future, CC could be supplemented with introductory teaching on DBT skills with follow up sessions to support practitioners in refining skills. Consideration also needs to be given to working in partnerships with professional training programmes of mental health practitioners. This involves focusing on core teaching to improve understanding of emotional dysregulation, and how mental health professionals can respond and work most effectively with such clients.

Several practical suggestions were made about the structure of the workshop. These minor suggestions included increasing font size on handouts and providing electronic copies of the presentation slides to improve the resources offered. One key point centred on the volume of information that was communicated over the course of the workshop. Specifically, practitioners felt that too much information was presented but recognised that it was necessary to convey all the content. Practitioners noted that they experienced difficulty concentrating during the latter part of the session. It is therefore recommended that the intervention is delivered in shorter but more frequent sessions to cover the existing CC programme, as well as the additional DBT skills. Another option is to develop supplementary online learning modules. This could facilitate the presentation of material in a more manageable format (e.g. a series of 30-min e-learning sessions) and also support skill strengthening. An adjunct training resource has been developed for the FC programme [26]. A parallel resource for practitioners may be of use.

### Limitations

There are a number of limitations to this study. Firstly, this is an uncontrolled, qualitative study. Quantitative research is now required to establish more robust evidence to support the initial themes identified by the analysis employed here. Secondly, the assumption was made that practitioners, by virtue of their professional training and other continuous professional development may need less input than families. Emerging themes indicate that it may be unwise to make such assumptions and the evidence presented here highlights that mental health practitioners have a need to increase their understanding of skills and support. Thirdly, the geographical location of the service and the study may have reduced the generalisability of the findings. In this geographical area, standard DBT and allied interventions have been available in the service for many years and consequently, many clinicians had some degree of familiarity with DBT. Therefore, the findings should be considered within the cultural context of an Irish population and the authors cannot confirm whether these findings will generalise to other contexts or cultures.

There are three components to FC: psychoeducation, skills training and support. CC attempted to mirror these functions. While CC certainly offered the opportunity for skills acquisition, qualitative feedback indicates that there is a strong case for additional CC sessions to support skills strengthening, consolidation and generalisation. While CC aimed to offer some support to clinicians and to establish a space where they could support and validate each other, there were natural limitations on the amount of support which could be realistically provided within the two CC sessions. It would be an important focus of further development of the CC programme to consider ways in which clinicians can be facilitated to support each other, and to facilitate them in establishing a peer support network.

### Future directions

While this initial qualitative study has been useful in providing an initial understanding of clinician experiences of CC, quantitative research is now necessary to quantify the impact of CC. In particular, gathering pre, post, and follow up data with a larger sample size in relation to constructs of skills acquisition/usage, work-related stress, and self-efficacy in working effectively with dysregulated clients will further our understanding in this area. A controlled comparison group comprising of practitioners working with this population who have not engaged in CC will be explored as a future research design.

## Conclusions

In conclusion, the initial evidence suggests that clinicians reported experiencing CC as feasible and beneficial. Skills such as validation and insight into BPD were outlined as useful in increasing practitioner confidence and improving relationships with clients. This strengthens the case for continuing to provide training in these areas to other practitioners within the public health service. It also suggests that an adapted FC programme can support mental health practitioners in meeting the needs of an individual with severe emotional dysregulation. Further training needs, in the form of understanding and use of some introductory DBT skills, have been identified to improve practitioners’ levels of self-efficacy and the quality of care provided to clients. These findings have implications for health service management who will need to consider how such continuing professional support needs can be facilitated. Based on the findings, the service in which the study was conducted has since implemented several changes to CC content and structure to better meet the needs of health practitioners. In light of this, further qualitative and quantitative research will be carried out to consider the ongoing utility of CC in supporting health care staff both understand and work more effectively with individuals presenting to services with severe emotional dysregulation.

## Data Availability

Larger samples of the qualitative data are available from the corresponding author upon reasonable request.

## Abbreviations

BPD: Borderline Personality Disorder
CC: Clinician connections
CMHTs: Community mental health teams
DBT: Dialectical behaviour therapy
ED: emergency department
FC: Family connections
SH: Self-harm.
